# High-performance extended-gate ion-sensitive field-effect transistors with multi-gate structure for transparent, flexible, and wearable biosensors

**DOI:** 10.1080/14686996.2020.1775477

**Published:** 2020-06-23

**Authors:** Jin-Hyeok Jeon, Won-Ju Cho

**Affiliations:** Department of Electronic Materials Engineering, Kwangwoon University, Seoul, Republic of Korea

**Keywords:** Extended-gate ion-sensitive field-effect transistor, polyethylene naphthalate, flexible, capacitive coupling, high-k engineered gate oxide, in-plane control gate, 208 Sensors and actuators, 306 Thin film / Coatings, 102 Porous / Nanoporous / Nanostructured materials

## Abstract

In this study, we developed a high-performance extended-gate ion-sensitive field-effect transistor (EG-ISFET) sensor on a flexible polyethylene naphthalate (PEN) substrate. The EG-ISFET sensor comprises a tin dioxide (SnO_2_) extended gate, which acts as a detector, and an amorphous indium-gallium-zinc-oxide (a-IGZO) thin-film transistor (TFT) for a transducer. In order to self-amplify the sensitivity of the pH sensors, we designed a uniquely-structured a-IGZO TFT transducer with a high-k engineered top gate insulator consisting of a silicon dioxide/tantalum pentoxide (SiO_2_/Ta_2_O_5_) stack, a floating layer under the channel, and a control gate coplanar with the channel. The SiO_2_/Ta_2_O_5_ stacked top gate insulator and in-plane control gate significantly contribute to capacitive coupling, enabling the amplification of sensitivity to be enlarged compared to conventional dual-gate transducers. For a pH sensing method suitable for EG-ISFET sensors, we propose an in-plane control gate (IG) sensing mode, instead of conventional single-gate (SG) or dual-gate (DG) sensing modes. As a result, a pH sensitivity of 2364 mV/pH was achieved at room temperature – this is significantly superior to the Nernstian limit (59.15 mV/pH at room temperature). In addition, we found that non-ideal behavior was improved in hysteresis and drift measurements. Therefore, the proposed transparent EGISFFET sensor with an IG sensing mode is expected to become a promising platform for flexible and wearable biosensing applications.

## Graphical abstract

**Figure:**
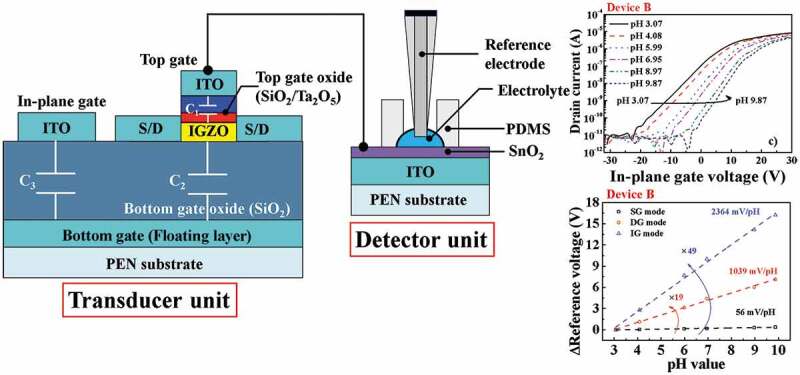

## 1. Introduction

Biological signals are often induced by small molecules, protons, and ions. Biosensors make it possible to monitor in situ the levels of these species, and to transduce the biological signal into an electrical signal that can be interpreted. The simplest signal in the cell, its pH level, can provide important information in cell membrane transport and other intracellular processes. At improper pH levels, biological cells’ functions can be damaged. pH is therefore fully controlled to ensure cell growth, proper cellular function, and normal intercellular processes [1,2]. Thus, monitoring and controlling pH in cells has significant potential for diagnostic and therapeutic applications [3]. Among various types of biosensors, electrical sensors as wireless wearable platforms represent the best opportunities for medical applications, due to their accurate detection, high sensitivity, low power consumption, fast response, and ease of signal processing [4]. Ion-selective electrodes are the most common electrical biosensors, but field-effect transistors (FETs) have matured into a versatile alternative; FETs excel in the continuous monitoring of small-scale analyte levels [5].

As traditional ion-sensitive field-effect transistors (ISFETs) have integrated sensing and transducer units, they have drawbacks in terms of their chemical instability and optical influence on sensitivity. In contrast, extended-gate ion-sensitive field-effect transistors (EG-ISFETs) can be reused by simply replacing the sensing unit, which is detachable from the transducer unit, when the sensing membrane is damaged. As the sensing unit is much cheaper to produce than the transducer unit, EG-ISFETs are suitable as disposable biosensors. However, both conventional EG-ISFETs and ISFETs are still subject having poor sensitivity (Nernst limit, 59.15 mV/pH at room temperature).

In addition, large arrays of EG-ISFETs are typically manufactured using silicon-on insulator (SOI) wafers for complementary metal-oxide-semiconductor (CMOS) fabrication [6]. Large-area EG-ISFETs are desirable because of their large ion-sensitive capture area, but fabricating them using SOI wafers can be prohibitively expensive, and requires high process temperatures. Thin-film transistors (TFTs) based on low temperature polycrystalline silicon (poly-Si) or metal oxide semiconductors, can be used to fabricate large-area liquid crystal displays (LCDs) and organic light emitting diode (OLED) display panels. They have been studied as a means to provide large-area device manufacturing at low costs [7]. In particular, metal oxide TFTs are expected to enable transparent, flexible, and wearable electronic device processing on substrates that are vulnerable at high temperatures [8].

In this study, we developed a high-performance EG-ISFET sensor for a transparent, flexible, and wearable biosensor platform, using a polyethylene naphthalate (PEN) substrate. As the EG-ISFET sensor constitutes both a detector and a transducer, we applied an extended gate consisting of a SnO_2_ sensing membrane and an amorphous indium-gallium-zinc-oxide thin-film transistor (a-IGZO TFT). Furthermore, we designed a highly efficient a-IGZO TFT transducer with an engineered SiO_2_/Ta_2_O_5_ stacked top gate insulator in the upper channel, a conductive floating layer under the channel, and a control gate in the same plane of the channel, to self-amplify the sensitivity of the pH sensors. To achieve a pH sensing method that is suitable for maximizing the sensitivity of the fabricated EG-ISFET sensor, we applied an in-plane control gate (IG) sensing mode, instead of conventional single-gate (SG) or dual-gate (DG) sensing modes. The increased capacitive coupling between the top gate and the in-plane control gate allowed the sensitivity to be greatly amplified, which was not attainable with conventional ISFETs or EG-ISFETs. In addition, to evaluate the stability and reliability of the pH sensors fabricated in electrolytes, we also tested their non-ideal behaviors, such as hysteresis and drift characteristics.

## 2. Methods

**Figure 1.**
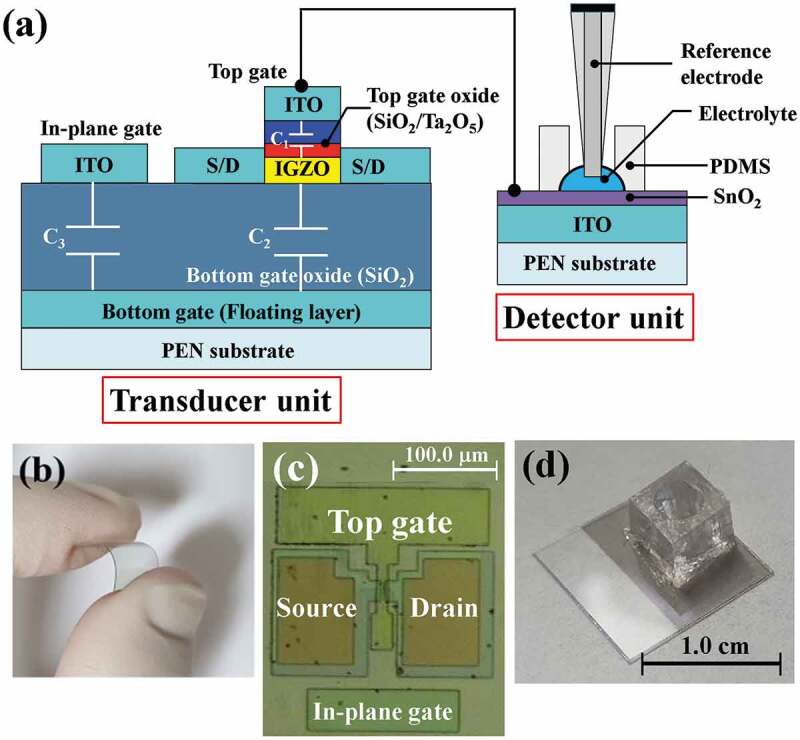
(a) Schematic 2D cross-sectional image of a fabricated EG-ISFET sensor, consisting of a high-k engineered top gate oxide (SiO_2_/Ta_2_O_5_), an in-plane control gate, and an EG with a SnO_2_ sensing membrane, (b) photograph and (c) optical microscope image of a fabricated in-plane control gate a-IGZO TFT transducer on a PEN substrate. (d) Photograph of an EG detector fabricated on a separate PEN substrate.

The EG-ISFET sensors fabricated in this study are composed of an a-IGZO TFT with an in-plane control gate and an extended gate with a SnO_2_ sensing membrane. These function as a transducer unit and a detector unit, respectively. The substrate material for a-IGZO TFT and EG was 125 μm-thick transparent PEN film. Prior to device processing, the PEN substrates were fixed on glass carriers for mechanical support, using a cool-off type adhesive tape (Intelimer tape CS2325NA4, Nitta Corp.). For the fabrication of the in-plane control gate a-IGZO TFT transducer, a 300-nm-thick indium tin oxide (ITO) layer and a 200-nm-thick SiO_2_ film were sequentially sputtered onto a PEN substrate as the bottom gate (floating layer) and the bottom gate oxide. A 20-nm-thick a-IGZO (In_2_O_3_:Ga_2_O_3_:ZnO = 2:1:2 mol.%) film was then deposited, followed by radio frequency (RF) magnetron sputtering. After the active region was formed by photolithography and wet etching, a 100-nm-thick ITO film was deposited by RF magnetron sputtering to form the source/drain (S/D) electrodes, using a lift-off process. Subsequently, a gate oxide consisting of either a 20-nm-thick single SiO_2_ layer (Device A) or a 10/35-nm-thick SiO_2_/Ta_2_O_5_ stacked layer (Device B) was deposited onto the active layer. The engineered top gate insulator, obtained by stacking SiO_2_ and Ta_2_O_5_, contributes not only to increasing the capacitive coupling, but also to reducing the gate leakage current. After depositing a 150-nm-thick ITO film by sputtering, the top gate and in-plane control gate electrode were simultaneously defined by photolithography and wet etching. The dimensions of the in-plane control gate were 100 × 20 μm, which were identical to the channel width and length. Finally, a post-metal annealing (PMA) was conducted to improve the contact properties of the ITO electrodes. In particular, because the PEN substrate is vulnerable to high temperatures, we suppressed substrate damage by annealing with 2.45 GHz microwave irradiation at 250 W for 2 min in H_2_/N_2_ (5/95 %, 50 sccm) in ambient conditions [9]. In addition, we prepared metal-insulator-metal (MIM) capacitors to determine the equivalent oxide thickness (EOT) of the top gate insulating layers. A 50-nm-thick Pt layer was used as the bottom electrode, a gate insulator (20-nm-thick single SiO_2_ layer or 10/35-nm-thick SiO_2_/Ta_2_O_5_ stacked layer), and a 150-nm-thick ITO film were used as the top electrode. These layers were sequentially deposited onto the substrate, using the same processes as for TFT fabrication. The ITO top electrode area of the MIM capacitor is 230 × 310 μm. Meanwhile, in order to fabricate the EG detector separately from the in-plane control gate, a-IGZO TFT transducer, a 300-nm-thick ITO film, and a 50-nm-thick SnO_2_ sensing membrane film were sequentially deposited using another PEN substrate. A 0.6 cm diameter polydimethyl siloxane (PDMS) reservoir (for electrolyte storage) was then fixed onto the ITO conductive layer. Figure 1(a) shows a schematic diagram of the EG-ISFET sensor, consisting of a high-k engineered top gate insulator (SiO_2_/Ta_2_O_5_), an in-plane control gate, and an EG with a SnO_2_ sensing membrane. Figure 1(b,c) show a photograph and an optical microscope image of the in-plane control gate a-IGZO TFT transducer, fabricated on a PEN substrate. Figure 1(d) shows a photograph of the EG detector with a SnO_2_ sensing membrane, fabricated on a separate PEN substrate. The capacitance-voltage (C-V) curve of the fabricated MIM capacitor was measured using an Agilent precision LCR meter 4284A  (HP, America), and the EOT was extracted according to the type of gate insulator. The characteristics of the EG-ISFET sensors were measured using an Agilent 4156B semiconductor parameter analyzer (HP, America). The Ag/AgCl reference electrode (Horiba 2080A-06 T with a ceramic-plug junction and an internal solution saturated with KCl and AgCl) was used to measure the pH sensitivity of the sensor, using various concentrations of pH buffer solutions. We investigated their reliability and stability by measuring non-ideal behaviors, such as hysteresis and drift effects, in the electrolyte. To avoid external influences, such as light and electrical noise, all measurements were conducted in a dark box.

## 3. Results and discussion

**Figure 2.**
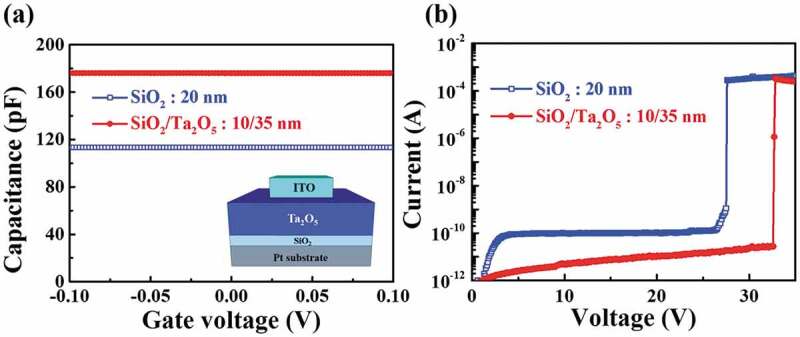
(a) capacitance-voltage (C-V) and (b) current-voltage (I–V) curves of the fabricated MIM capacitors, with either a 20-nm-thick single SiO_2_ layer or a 10/35-nm-thick SiO_2_/Ta_2_O_5_ stacked layer.

The capacitive coupling effect is an important consideration when designing high sensitivity in-plane control gate a- IGZO TFT transducers. In particular, the top gate insulator above the TFT channel determines the amplification factor of the transducer. This is achieved through capacitive coupling with the bottom gate insulator, below the TFT channel. Thinner top gate insulators and thicker bottom gate insulators are effective in increasing capacitive coupling, leading to higher sensor sensitivity. Figure 2 shows the C-V and current-voltage (I–V) curves of the fabricated MIM capacitors. Capacitor B (10/35-nm-thick SiO_2_/Ta_2_O_5_ stacked layer) has a larger capacitance than capacitor A (20-nm-thick single SiO_2_ layer), as shown in Figure 2(a). The EOT of capacitor B, calculated from the measured capacitance, is 13.34 nm, which is thinner than that of capacitor A. In addition, as shown in Figure 2(b), the engineered gate insulator, in which a 10-nm-thick SiO_2_ layer and 35-nm-thick Ta_2_O_5_ are stacked, has a lower leakage current and a larger breakdown voltage than a 20-nm-thick SiO_2_ single gate insulator.

**Figure 3.**
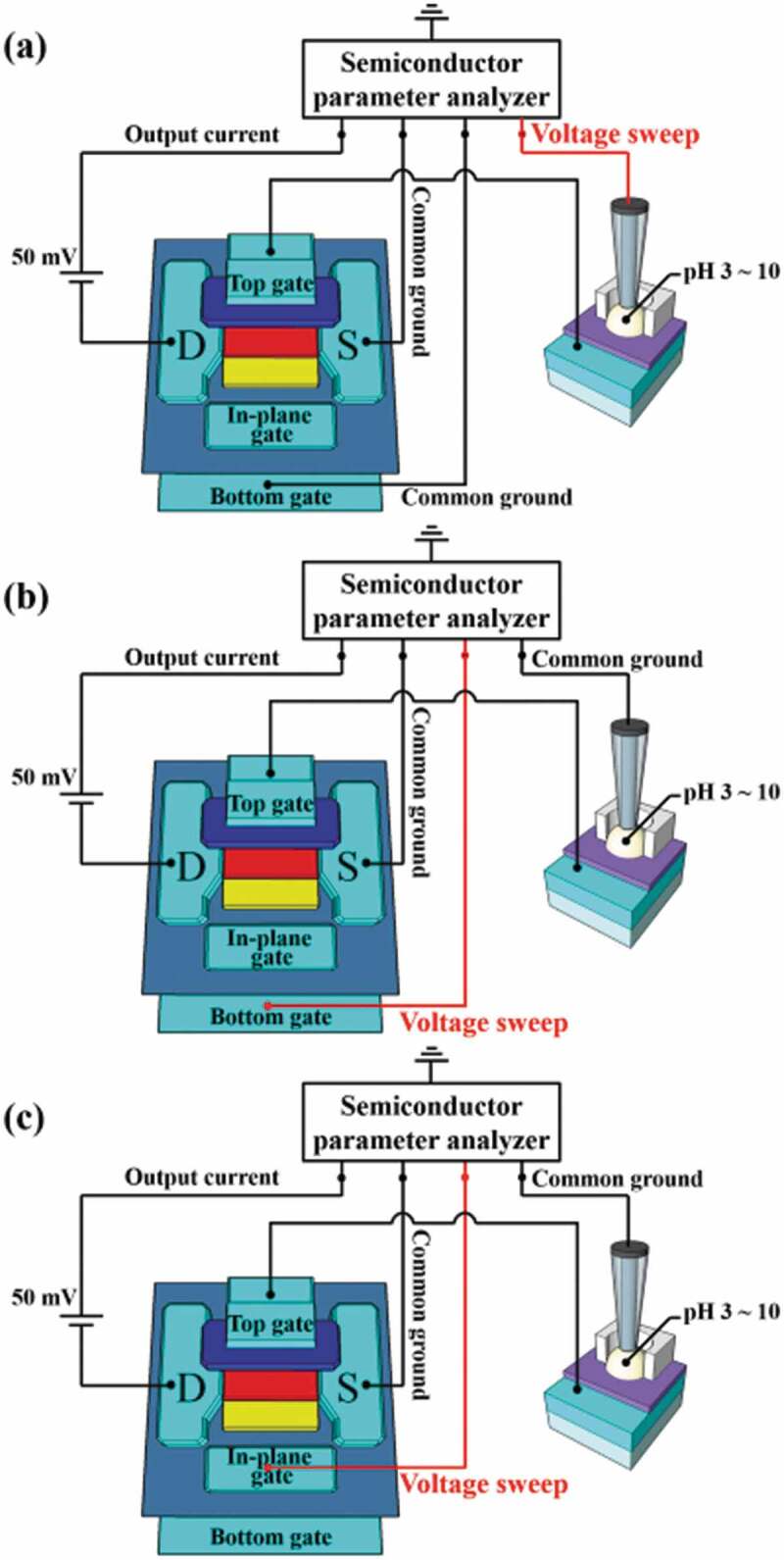
Operating modes of the EG-ISFET sensors with high-k engineering oxide and in-plane control gates: (a) SG, (b) DG, and (c) IG sensing modes.

Figure 3 shows the three modes of operation for an EG-ISFET sensor consisting of an in-plane control gate, a-IGZO TFT transducer unit, and an EG detector unit. The first mode the SG mode sensing, which uses only the top gate at the top of the channel. In this case, the BG electrode is grounded, and is voltage-scanned (single-gate voltage: V_SG_) to the top reference electrode to measure drain current, as shown in Figure 3(a). This is used in conventional SG structure ISFETs. The second mode, DG mode sensing, measures the drain current while grounding the top reference electrode and voltage-scanning (bottom-gate voltage: V_BG_) with the BG electrode, as shown in Figure 3(b). The third mode is IG mode sensing, which measures the drain current while grounding the top reference electrode and voltage-scanning (in-plane-gate voltage: V_IG_) with the in-plane control gate electrode, as shown in Figure 3(c).

**Figure 4.**
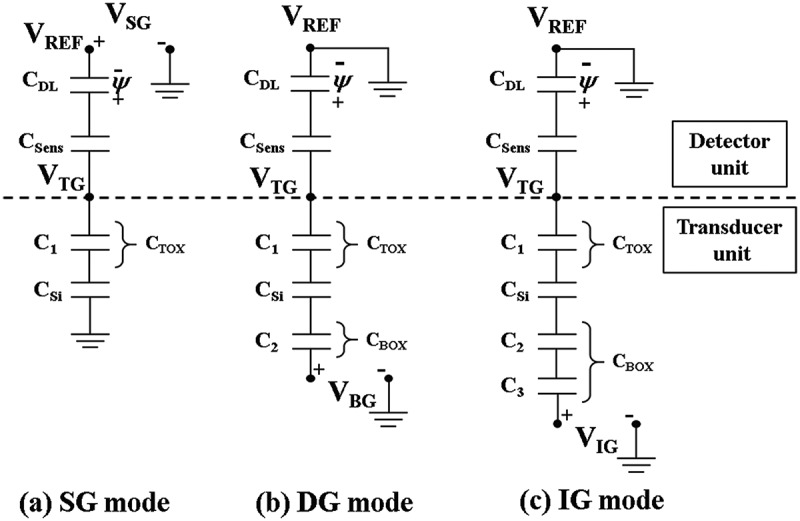
Equivalent circuits for the sensing modes of the EG-ISFET sensor: (a) SG, (b) DG, and (c) IG. C_1_ is the top gate insulator capacitance, C_2_ is the bottom gate insulator capacitance, C_3_ is the in-plane gate insulator capacitance, C_IGZO_ is the a-IGZO depletion capacitance, and C_Sens_ is the sensing membrane capacitance.

Figure 4 shows the equivalent circuits for the three sensing modes of the fabricated EG-ISFET sensors. C_1_ is the top gate insulator capacitance, C_2_ is the bottom gate insulator capacitance, C_3_ is the in-plane gate insulator capacitance, C_IGZO_ is the a-IGZO depletion capacitance, and C_Sens_ is the sensing membrane capacitance. The C_Sens_ is so large that it is not considered. The surface potential (ψ) represents the pH level of the electrolyte, and is strongly dependent on the nature of the sensing membrane. The V_th_ of the ISFETs is shifted according to the surface potential (∆V_th_ = -∆*ψ*). The theoretical maximum value of the pH sensitivity in the SG mode sensing in Figure 4(a) is limited to the Nernst limit (59.15 mV/pH at room temperature) by the site binding model. Furthermore, the ion sensing ability depends on the surface potential [10]. In the DG mode, as capacitive coupling occurs between the top gate and the bottom gate insulators, as shown in Figure 4(b), the capacitive coupling ratio and the sensitivity can be described by the following simplified Equation (1) [11]:

(1) $$\Delta V_{BG}^{Th}=-\frac{C_{top}}{C_{bottom}}\Delta \psi =\frac{C_{1}}{C_{2}}\Delta V_{TG}^{Th}$$

where, C_1_ and C_2_ are the capacitances between the top gate and the channel, and between the channel and the bottom control gate, respectively. $V_{BG}^{Th}$ and $V_{TG}^{Th}$ are the threshold voltage shifts of the bottom control gate and top sensing gate, respectively. The potential change of the top sensing gate shifts the threshold voltage shift of the bottom control gate, in proportion to C_1_/C_2_. To increase the coupling ratio and the sensitivity, the DG ISFET should have a thicker bottom gate insulator, or a thinner top gate insulator. However, a thicker bottom gate insulator requires longer processing times and higher costs. A thinner top gate insulator results in an increase in gate leakage current, which is considered an inefficient approach. The EG-ISFET (with a high-k engineering top gate insulator and in-plane control gate) proposed in this study can self-amplify the sensitivity more efficiently in the IG mode. As shown in Figure 4(c), the in-plane control gate has the effect of doubling the thickness of the bottom gate oxide [12]. The capacitive coupling ratio and the sensitivity amplification in IG mode can then be described as follows in Equation (2):

(2) $$\Delta V_{IG}^{Th}=-\frac{C_{top}}{C_{bottom}}\Delta \psi =\frac{(C_{2}+C_{3})C_{1}}{\left(C_{2}\times C_{3}\right)}\Delta V_{TG}^{Th}$$

where, C_1_, C_2_, and C_3_ are the capacitances between the top gate and the channel, between the channel and the floating layer, and between the floating layer and the in-plane control gate, respectively. $V_{IG}^{Th}$ is the threshold voltage shift of the in-plane control gate. The IG mode exhibits more benefits than a DG mode for increasing capacitive coupling, because C_2_ and C_3_ are connected in series. The potential change of the top sensing gate shifts the threshold voltage shift of the in-plane control gate, in proportion to (C_2_+ C_3_)C_1_/(C_2_× C_3_).

**Figure 5.**
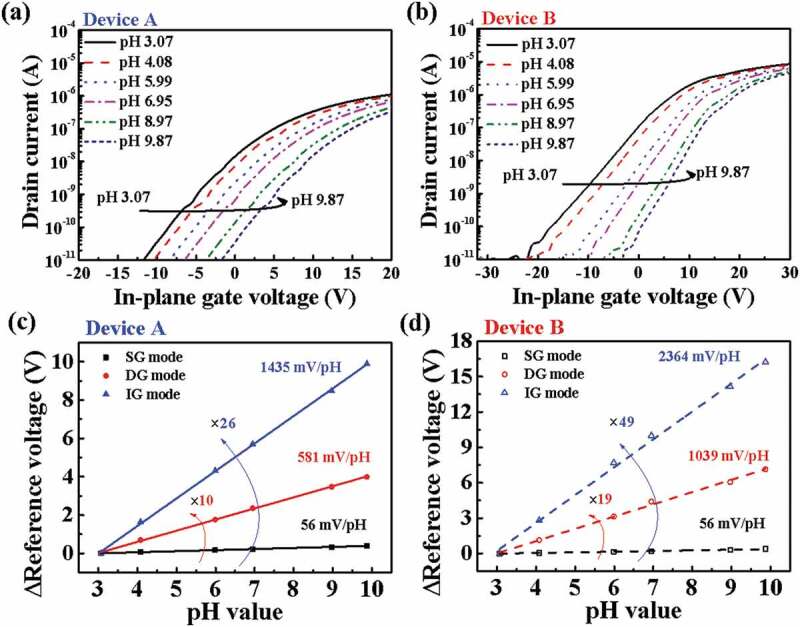
Typical transfer curves of EG-ISFET sensors with (a) Device A and (b) Device B transducers, according to pH concentration (IG mode sensing). Sensitivity of (c) Device A and (d) Device B, according to SG, DG, and IG sensing modes.

Figure 5 shows the transfer curves and pH sensitivities of the EG-ISFETs. Typical transfer curves in the IG mode sensing for (a) Device A and (b) Device B are shown in Figure 5(a,b), respectively. When the pH electrolyte solution changes the surface potential of the sensing membrane according to the pH concentration, it is transferred to the top sensing gate. As a result, the threshold voltage of the in-plane control gate voltage increases with increasing pH. In this study, we defined the reference voltage (V_R_) instead of the threshold voltage, as the control gate voltage at drain current I_DS_ = 1 nA, and we defined the amount of V_R_ shift (ΔV_R_) per unit of pH as the sensitivity. The pH sensitivity of the EG-ISFETs under SG, DG, and IG modes are shown in Figure 5(c,d). In SG mode, the sensitivities of Devices A and B both were 56 mV/pH, respectively. These values are close to the Nernst limit set by the site-bound surface. In the DG mode, the sensitivities of Devices A and B were amplified to 581 and 1039 mV/pH, respectively, depending on Equation (1). Meanwhile, the IG mode sensing can amplify the sensitivity to a much greater extent than the DG mode sensing, according to Equation (2). We were able to achieve very high sensitivities (1435 and 2364 mV/pH) using the IG mode for Devices A and B, respectively. The highest pH sensitivity was obtained under the IG mode of the EG-ISFETs for Device B.

**Figure 6.**
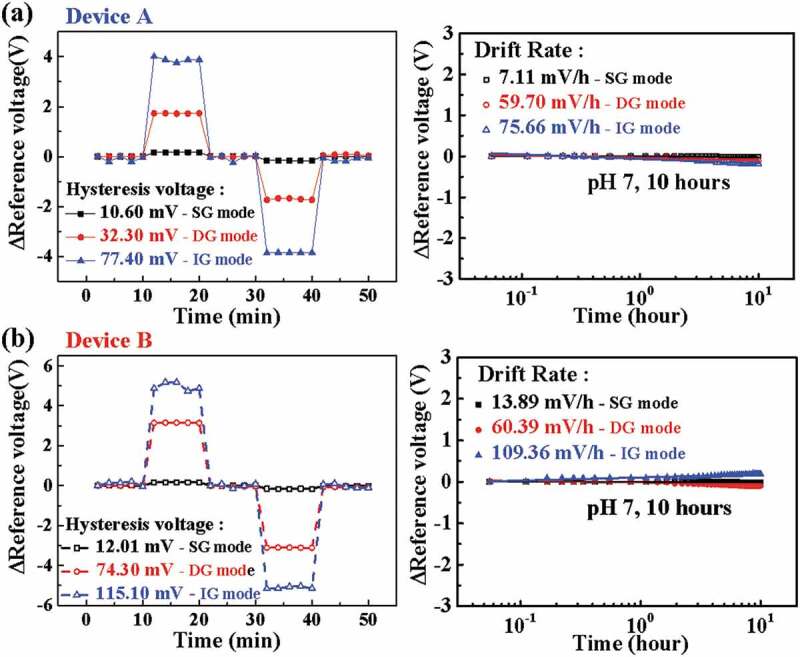
Hysteresis and drift rate of EG-ISFET sensors with (a) Device A and (b) Device B transducers for evaluation of stability and reliability, according to SG, DG, and IG sensing modes.

Figure 6 shows the stability of the EG-ISFET sensors with Device A or B transducers. This was evaluated by measuring the hysteresis voltage and drift rate in the electrolyte solution. The hysteresis voltage shown in Figure 6(a) is caused by the micro-charging of the sensing membrane, which occurs when ions in the electrolyte solution react slowly with the surface of the sensing membrane. We defined the hysteresis voltage as the change between the V_R_ of the first pH 7 solution and the V_R_ of the last pH 7 solution in the pH loop (7 → 10 → 7 → 4 → 7) [13].

In addition, the drift rate shown in Figure 6(b) represents the difference in V_R_ per hour due to micro-charging, as ions penetrate into the sensing membrane over a long period of time [14]. The drift rates of Device A under the SG, DG, and IG modes were 7, 60, and 76 mV/h, respectively. On the other hand, for Device B, the drift rates were 14, 60, and 109 mV/h under the SG, DG, and IG modes, respectively. When we take hysteresis and drift rate into consideration regarding sensitivity, Device B in IG mode is believed to have the best stability against drift effects (summarized in Table 1). As a result, it can be concluded that the proposed EG-ISFETs have good stability in the IG mode. The pH sensing characteristics of the EG-ISFETs with high-k engineered oxides and in-plane control gates are summarized in Table 1.

**Table 1.** pH sensing characteristics of EG-ISFETs with high-k engineered top gate insulators and in-plant control gates.

Device	Operation mode	Sensitivity (mV/pH)	Hysteresis (mV)	Drift rate (mV/hour)	Hysteresis for sensitivity (%)	Drift rate for sensitivity (%)
A	SG	56	11	7	19	13
	DG	581	32	60	6	10
	IG	1435	77	76	5	5
B	SG	56	12	14	22	25
	DG	1039	74	60	7	6
	IG	2364	115	109	5	5

## 4. Conclusions

In this study, we have developed high-performance EG-ISFET sensors for transparent, flexible and wearable biosensor platforms, using PEN substrates. These EG-ISFET sensors have a SnO_2_ extended gate as a detector and an a-IGZO TFT as a transducer. In order to self-amplify the sensitivity of these pH sensors, we designed a uniquely-structured a-IGZO TFT transducer with a high-k engineered top gate insulator consisting of a SiO_2_/Ta_2_O_5_ stack, an electrically isolated floating layer, and an in-plane control gate. Regarding the pH sensing method suitable for EG-ISFET sensors, we proposed an IG sensing mode instead of the conventional SG or DG sensing modes. In the IG sensing mode, the SiO_2_/Ta_2_O_5_ stacked top gate insulator increased the capacitance of the top gate insulator, whereas the capacitance of the bottom gate was reduced almost by half. This acted to maximize the sensitivity amplification. As a result, we achieved a pH sensitivity of 2364 mV/pH at room temperature, far beyond the Nernstian limit (59.15 mV/pH at room temperature). In addition, non-ideal behavior was improved in hysteresis and drift measurements; by considering the sensitivity, we were able to reduce the noise of the proposed sensors. Therefore, the proposed transparent EGISFFET sensor (in the IG sensing mode) is expected to be a promising platform for flexible and wearable biosensing applications, as it demonstrates high sensitivity and excellent stability.

## References

[1] Flinck M, Kramer SH, Pedersen SF. Roles of pH in control of cell proliferation. Acta Physiol. 2018;223(3):e13068. 10.1111/apha.13068 29575508

[2] Boron WF. Regulation of intracellular pH. Adv Physiol Educ. 2004;28(4):160–179. 15545345 10.1152/advan.00045.2004

[3] Pavlov I, Kaila K, Kullmann DM, et al. Cortical inhibition, pH and cell excitability in epilepsy: what are optimal targets for antiepileptic interventions? J Physiol. 2013;591(4):765–774. 22890709 10.1113/jphysiol.2012.237958 PMC3591695

[4] Ronkainen NJ, Halsall HB, Heineman WR. Electrochemical biosensors. Chem Soc Rev. 2010;39(5):1747–1763. 20419217 10.1039/b714449k

[5] Chen K-I, Li B-R, Chen Y-T. Silicon nanowire field-effect transistor-based biosensors for biomedical diagnosis and cellular recording investigation. Nano Today. 2011;6(2):131–154.

[6] Meyburg S, Goryll M, Moers J, et al. N-Channel field-effect transistors with floating gates for extracellular recordings. Biosens Bioelectron. 2006;21(7):1037. 16029948 10.1016/j.bios.2005.03.010

[7] Estrela P, Keighley SD, Li P, et al. Application of thin film transistors to label-free electrical biosensors. IEEE Int Symp Ind Electron. 2008;14(4):2034.

[8] Smith JT, Shah SS, Goryll M, et al. Flexible ISFET biosensor using IGZO metal oxide TFTs and an ITO sensing layer. R IEEE Sensors J. 2014;14(4):937.

[9] Hong EK, Cho WJ. Effect of microwave annealing on SOI MOSFETs: post-metal annealing with low thermal budget. Micron Reliab. 2018;80:306–311.

[10] Garrido JA, Härtl A, Kuch S, et al. pH sensors based on hydrogenated diamond surfaces. Appl Phys Lett. 2005;86:073504.

[11] Park JK, Jang HJ, Park JT, et al. SOI dual-gate ISFET with variable oxide capacitance and channel thickness. Solid State Electron. 2014;97:2.

[12] Jang HJ, Bae TE, Cho WJ. Improved sensing performance of polycrystalline-silicon based dual-gate ion-sensitive field-effect transistors using high-k stacking engineered sensing membrane. Appl Phys Lett. 2012;100(25):253703.

[13] Tsai CN, Chou JC, Sun TP, et al. Study on the sensing characteristics and hysteresis effect of the tin oxide pH electrode. Sens Actuators B. 2005;108(1–2):877.

[14] Jamasb S, Collins S, Smith RL. e. A physical model for drift in pH ISFETs. Sens Actuators B. 1998;49(1–2):146.

